# Performance, Nutrient Digestibility and Physiological Resilience of Juvenile Gilthead Seabream (*Sparus aurata*) Fed Organic and Circular Economy-Derived Diets

**DOI:** 10.1155/anu/9559268

**Published:** 2025-08-19

**Authors:** Rodrigo Mendes, Rita Teodósio, Jorge Dias, Ana Teresa Gonçalves, Lais Speranza, Sara Magalhães, Tiago Aires, Francisco J. Sánchez-Vázquez, Luís E. C. Conceição, Sofia Engrola

**Affiliations:** ^1^Sparos Lda., Olhão, Portugal; ^2^Universidade do Algarve, Centro de Ciências do Mar, Faro, Portugal; ^3^Universid de Murcia, Facultad de Biologia, Murcia, Spain; ^4^GreenCoLab, Faro, Portugal; ^5^Sorgal, S. João de Ovar, Portugal

**Keywords:** aquafeeds, fish performance, fish resilience, gilthead seabream, global warming potential

## Abstract

Aquafeeds formulated with organic or circular economy-derived ingredients aim to enhance sustainability and consumer acceptance. This study evaluated the global warming potential (GWP) and digestibility of such feeds, and assessed their effects on performance, feed utilisation and physiological resilience, defined as the ability to maintain tissue function and integrity under different feeding conditions of juvenile gilthead seabream (*Sparus aurata*) during grow out and after an overcrowding stress challenge. Three isonitrogenous (~51% crude protein) and isoenergetic (~18% crude fat) diets with limited fishmeal were formulated: a control (CTRL) commercial-like feed; an organic (ORG) diet based on organic-certified ingredients rich in plant proteins (primarily pea protein concentrate and wheat gluten); an eco-efficient (ECO) diet mainly composed of circular economy-derived animal by-products (e.g., poultry meal and feathermeal hydrolysate). The GWP was estimated using a life cycle assessment. Juvenile seabream (~14 g) were stocked in triplicate 500 L tanks (90 fish per tank, initial density of 2.5 kg/m^3^) and fed three times daily following feeding tables generated by FiT Feeding Tables, to optimise ration and minimise waste, over a 65-day growth period (final density of 8 kg/m^3^) and a subsequent 14-day overcrowding challenge (initial density of 12.4 kg/m^3^). At the end of the growth period, all groups exhibited at least a threefold increase in body weight. Feed digestibility was high (apparent digestibility coefficients (ADCs) > 60%) and utilisation efficient. Physiological resilience was supported by stable growth and relative expression of biomarkers for gut health, oxidative status and immune function. Although ORG and ECO diets showed a higher GWP, this impact may decrease with increased use of renewable energy in ingredient production. The ORG diet also improved fish phosphorus retention. These organic and circular economy-derived feeds present viable options to reduce aquaculture's environmental footprint while maintaining fish performance and resilience.

## 1. Introduction

Nowadays, society is more demanding with the sustainability of food and aquaculture products [1–4]. Consumers are particularly concerned with the environmental impacts and food safety (e.g., the organic food movement), as well as carbon footprints, alongside the health and welfare of farmed aquatic animals [5, 6]. In response, aquaculture must promote its societal perception, improve its environmental performance and enhance farming conditions [7]. These aligns with the objectives of the European Union (EU), which aim to increase aquaculture production, by focusing on food safety, ethical standards and especially environmental sustainability [8–10].

Aquafeeds, a major contributor to the environmental footprint of aquaculture, are a critical area for improvement in environmental sustainability. There has been some progress to reduce the reliance and environmental footprint of commonly used feed ingredients (e.g., marine and soy-based sources), mainly through the implementation of responsible management and sourcing practices, certification schemes, regulatory frameworks and technological innovations [11, 12]. However, the progress in adopting environmentally sustainable practices varies across different regions. Accordingly, aquafeeds production continues to play a significant role in contributing to the industry's environmental impacts, particularly in terms of resource consumption and global warming [12–15]. Feed ingredients can represent around 60%–70% of the carbon emissions of seafood farming [16]. These figures underscore the need for the development of alternative feed formulations that may reduce some of aquaculture environmental impacts.

Alternative feed ingredients sourced from organic production systems or developed within circular economy frameworks, such as those based on the valorisation of by-products and waste streams, are generally well perceived by society and may contribute to improving the environmental sustainability of aquaculture. Organic formulations address ethical and food safety concerns, while circular economy feed concepts may reduce forage fish demand, promote overall system performance and enhance resource efficiency [17–19]. However, these advantages, particularly regarding the global warming potential (GWP), vary significantly from production sites and need a thorough evaluation.

The GWP is a key metric to indicate the environmental impacts of aquafeeds, reflecting their contribution to climate change through carbon or greenhouse gas (GHG; e.g., CO_2_) emissions [20]. The GWP can be assessed through a life cycle assessment (LCA), similar to what has been done by Basto-Silva et al. [21] and Bergman et al. [22] with gilthead seabream (*Sparus aurata*) and rainbow trout (*Oncorhynchus mykiss*) feeds, respectively. This assessment is especially relevant when feed formulations are based on distinct types of alternative ingredients.

Potential alternative ingredients must meet several key requirements: wide availability, promote good fish performance, have a low environmental footprint and ensure animal welfare. Suitable alternatives could include land animal by-products (LAPs; e.g., blood-, feather- and poultry meals), single cell microorganisms (SCMs; e.g., microalgae, yeast and bacteria) and some plants (e.g., potato, sunflower and rapeseed). LAPs are a subset of processed animal proteins (PAPs), derived exclusively from non-aquatic animal by-products, whereas PAPs include both terrestrial and aquatic animal sources [23]. LAPs are affordable, widely available and can reduce waste generation while valorising side streams [11, 24–26]. SCMs have rapid growth, can be intensively produced year-round and its cultivation systems are amenable to a high degree of automation [27, 28]. Methods, infrastructure and systems for large-scale production and processing of plants are already well-established [11]. However, aquafeed formulations must be examined on a case-by-case basis. This is especially important since some ingredients and combinations, at certain inclusion levels, may result in adverse effects, such as reduced feed intake (FI), depleted growth or immune disturbances caused for example by antinutritional factors (ANFs; solanine, tannins and phytates), commonly present in plants [29–33]. Particular attention should be given to the intestine, as the organ is the primary sensor of dietary changes given its key role in fish digestion/metabolism [29, 30]. This is especially relevant in commercially important species, such as gilthead seabream (*Sparus aurata*), a pivotal farmed fish part of the gastronomic culture and seafood economy of the Mediterranean, with an annual global production of approximately 280 thousand metric tonnes [34, 35].

This study aimed to evaluate the digestibility and effects of feeds formulated within organic or circular economy-derived (eco-efficient) frameworks on the performance, feed utilisation and physiological resilience of juvenile gilthead seabream (*Sparus aurata*) under grow out conditions. In this context, resilience refers to the fish's capacity to maintain organ function and overall health status under dietary and environmental challenges. At the end of the growth period, fish were exposed to a chronic stressor (overcrowding) to assess their resilience. The GWP of the feeds was also evaluated.

## 2. Materials and Methods

### 2.1. Experimental Diets

Three experimental diets: control (CTRL), organic (ORG) and eco-efficient (ECO) were formulated and produced by SPAROS Lda (Olhão, Portugal). Powder ingredients were mixed accordingly to a target formulation in a double-helix mixer (model 500 L, TGC Extrusion, Roullet-Saint-Estèphe, France) and grounded (below 2.0 mm) in a micropulverizer hammer mill (model SH1, Hosokawa-Alpine, Augsburg, Germany). Diets (pellet size: 2.0 mm) were extruded with a twin-screw extruder (model BC45, Clextral, Firminy, France) and dried in a convection oven (OP 750-UF, LTE Scientific, Oldham, UK). After cooling, oils were added to the pellets by vacuum coating (model PG-10VCLAB, Dinnisen, Sevenum, The Netherlands). Throughout the duration of the study, experimental diets were stored inside plastic buckets at room temperature in a cool and aerated storage room. Representative samples from each diet were collected and analysed for proximate composition and amino acid profile analyses.

The formulation concept and ingredient selection (Table 1) was based within an organic or circular economy framework, on market availability and nutritional composition to fulfil the known nutritional requirements of juvenile gilthead seabream [36–39]. The control feed (CTRL) was formulated to mimic a commercial formulation, being soy-free and with medium levels of LAPs. The organic (ORG) feed was primarily designed to limit the inclusion of fishmeal and LAPs, which were replaced with plant sources (e.g., pea protein, potato protein, rapeseed meal and oil). In addition, the ORG diet included SCMs (microalgae and yeast) along with other ingredients compatible with organic production standards and available on the organic market. Although not fully composed of certified organic ingredients, diet ORG was formulated to reflect organic principles. Here, ‘organic-based' refers to the relatively higher use of organic-compatible ingredients, rather than full compliance with EU organic certification. The eco-efficient (ECO) feed was formulated with ingredients similar to those used in the CTRL feed, but with higher inclusion levels of LAPs (feathermeal hydrolysate, poultry meal and poultry blood meal) and limited fishmeal. All diets were formulated to be isonitrogenous (crude protein of ~51% as fed), isoenergetic (gross energy of ~22.2 kJ/g as fed; Table 1) and with similar fatty acid profiles (estimated 2.2% of eicosapentaenoic + docosahexaenoic acids (EPA + DHA), based on ingredient analysis). The GWP of each feed is presented in Table 1. The dietary treatments (CTRL, ORG and ECO) were randomly assigned to replicate tanks (*n* = 3 replicates per dietary treatment).

Experimental diets were analysed for total amino acid content according to Aragão et al. [29]. In brief, samples underwent acid hydrolysis (6 M HCl at 116°C for 48 h in nitrogen-flushed glass vials) and were then pre-column derivatised with Waters AccQ Fluor Reagent (6-aminoquinolyl-N-hydroxysuccinimidyl carbamate) using the AccQ Tag method (Waters, Milford, MA, USA). Analyses were done by ultra-high-performance liquid chromatography (UPLC) in a Waters reversed-phase amino acid analysis system, using norvaline as an internal standard. The resulting chromatograms were analysed with EMPOWER software (Waters, USA). Amino acid profiles are presented in Table 2.

### 2.2. LCA

GWP was calculated using the LCA methodology with economic allocation. Agribylase (ADEME, France), Ecoinvent (Zürich, Switzerland) and Global Feed LCA Institute (GFLI; Zoetermeer, The Netherlands) databases, as well as information present in literature and CarbonCloud (https://www.carboncloud.com CarbonCloud, Göteborg, Sweden) were used as sources of background data regarding raw materials. Although this data can present some level of uncertainty, the most accurate information was used. For each ingredient, the GWP value obtained from the databases was multiplied by the inclusion level of that ingredient in the diet. The GWP contributions of all ingredients were then summed to calculate the total GWP of each diet. The system boundary was set to include the grow out, fishery activities or production of feed ingredients (including energy usage) as well as processing and transportation from production to processing locations and final product from factory to markets. Ingredient mixing and pelletization were not considered.

### 2.3. Fish Husbandry

#### 2.3.1. Fish Feeding

Before starting the experiment, a tailored feeding table was generated according to the species requirements, with the goal of avoiding overfeeding, ensure good fish performance and optimum experimental conditions (e.g., appropriate oxygen saturation levels) during summer with rapidly growing juveniles. This table served as a guideline to determine the maximum feed that would be given, by hand, to each fish tank daily. At times, smaller quantities were given when fish did not show appetite for the full estimated ration. The FiT Feeding Tables (https://www.sparos.pt/products/#fit) tool developed by SPAROS and RIASEARCH was used. As input data, the experimental conditions (fish body weight and water temperature) from a previous study [40] performed in the same fish facilities and season (summer) with gilthead seabream were considered. This choice was made because the experimental conditions were expected to be similar to those of the present trial. Data on diet proximate composition and digestibility were defined based on the feeds used. Subsequently, the table was generated using the energy and protein fluxes (EP model) defined by Nobre et al. [41] and derived from the bioenergetic factorial approach [36]. The same table was used for all feeds (CTRL, ORG and ECO) since the differences in proximate composition were marginal. The daily feeding rate values obtained were then applied to the thermal growth coefficient (TGC) model, using the formula provided by Besson et al. [42], in order to estimate fish growth and, thereby determine the amount of feed that would be given to each tank daily. In addition, the TGC model is based on the relationship between water temperature and fish weight allometry [43]. To update the TGC model enabling more precise calculations of the feed to give to each tank, water temperature was measured daily and two intermediate fish bulk weight measurements (on days 19 and 40) were performed to monitor fish growth and accurately reflect the biomass in each tank. Fish were patiently fed by hand the calculated amounts of one of the experimental diets (CTRL, ORG or ECO), using the previously generated feeding table as guideline, three times per day (in equal portions) from Monday to Saturday (09:45, 11:45 and 16:00) and twice on Sundays (09:45 and 11:45). If fish were satisfied before all the predetermined feed was given, the leftover weight was recorded.

#### 2.3.2. Growth Period

The trial was carried out at the Ramalhete Experimental Research Station of the Centre of Marine Sciences of Algarve (CCMAR, Faro, Portugal). Trained scientists performed the trial, following the European Directive 2010/63/EU of European Parliament and of the Council of EU on the protection of animals used for scientific purposes, being approved by the Committee of Ethic and Animal Experimentation of CCMAR. The CCMAR facilities and their staff are certified to house and conduct experiments with live animals (‘Group-1' licence by the ‘Direção Geral de Veterinaria', Ministry of Agriculture, Rural Development and Fisheries of Portugal).

Gilthead seabream (*Sparus aurata*) juveniles were supplied from a commercial farm and transported to the to the research station by an authorised carrier. No mortality or pathological signs were observed in association to transport. During the acclimation period of 3 weeks, fish were fed twice a day using a commercial diet (Standard 4 Orange, Sorgal, Aveiro, Portugal; 43% CP, 17% CF, according to manufacturer data).

At the start of the trial, treatments were randomly assigned to tanks. A total of 810 gilthead seabream juveniles (90 fish per tank) with a mean body weight of 14.1 ± 0.02 g (mean ± SD) were progressively distributed into nine 500 L outdoor cylindrical fibreglass tanks (initial density 2.5 kg/m^3^). To ensure homogeneous initial biomass (CV < 5%) across tanks, fish were counted and weighed in small groups of five during allocation, allowing real-time adjustment of total fish weight per tank. After being fasted for 24 h, 15 fish from the initial stock were euthanised with a lethal dose of anaesthetic (1000 mg/L; 2-phenoxyethanol, Sigma–Aldrich, Spain), pooled and stored at −20°C for subsequent analysis of whole-body composition. From the initial stock, 42 fish, that resembled the experimental population, were measured and weighed individually to obtain the initial condition factor. Tanks were supplied with flow-through, gravel-filtered, aerated seawater and subjected to natural photoperiod changes through summer conditions (May–July). Abiotic parameters (temperature: mean 23.5 ± 2.1°C, ranging from 27.3 to 18.8°C; salinity: 37.8 ± 0.4 ‰; oxygen saturation: 96.3% ± 1.4%), FI and mortality were measured and recorded daily. The growth period lasted for 65 days. At the end of the growth period, fish were fasted for 24 h, counted and bulk weighted to monitor growth performance, feed utilisation and retention indicators. Additionally, 12 fish per replicate tank (*n* = 36 fish per dietary treatment) were euthanised, sampled, individually measured and weighted, before their viscera and liver were carefully sampled and weighed for determination of the somatic indices. Of these, six fish per replicate tank (*n* = 3 pools per dietary treatment) were pooled and frozen at −20°C for analysis of whole-body composition. In addition, six fish from each replicate tank (*n* = 18 fish per dietary treatment) were dissected for the anterior intestine. A small section of the tissue was preserved in RNA later (Sigma–Aldrich, Madrid, Spain) until analysed for gene expression. All samples were kept at −80°C until further analysis.

#### 2.3.3. Challenge Period

At the end of the growth period, after sampling fish (e.g., whole-body composition, genetic analysis, somatic indices and to balance the number of fish among all tanks), an average of 67 fish remained in each tank (*n* = 201 fish per dietary treatment). These fish, with an initial body weight of 46.4 ± 3.0 g (obtained after counting and bulk weighing at the end of the growth period), were then exposed to chronic overcrowding stress for 2 weeks using the same experimental setup. This was achieved by decreasing the tank water volume to 250 L, consequently increasing the density to 12.4 kg/m^3^ (from 8 kg/m^3^ at the end of the growth period). The feeding regimes were kept, and fish were fed similarly to the growth period. Abiotic parameters (temperature: 23.9 ± 0.9°C; salinity: 38.4 ± 0.4‰; oxygen saturation: 93.7% ± 1.8%), FI and mortality were measured and recorded daily. At the end of the challenge period, after fasting for 24 h, all fish on each tank were counted and bulk-weighted for key performance indicators. In addition, six fish from each replicate tank (*n* = 18 fish per dietary treatment) were euthanised with a lethal dose of anaesthetic (1000 mg/L; 2-phenoxyethanol, Sigma–Aldrich, Spain) and dissected for the anterior intestine for gene expression analysis, following the same procedure used at the end of the growth period.

### 2.4. Digestibility Trial

Nine conical fibreglass tanks (100 L; *n* = 3 per dietary treatment) were stocked with homogeneous groups of 20 gilthead seabream juveniles (~25 g each), selected from the initial stock. Fish were hand-fed the experimental diets (CTRL, ORG, and ECO) twice daily (09:45 and 12:00) to apparent satiation for 3 weeks. This feeding routine was selected to maximise faecal output and ensure adequate sample collection for reliable digestibility assessment, rather than to evaluate growth performance. Feeding to apparent satiation is considered the most appropriate and practical strategy for digestibility trials, particularly when aiming to reflect commercial conditions and ensure sufficient faeces production [44]. Additionally, the standardised method of faecal collection used in this study minimises the risk of underestimating digestibility, commonly associated with techniques such as stripping, while also reducing variability [44]. The extended collection period and large number of fish per treatment contribute to improved data robustness and greater reliability of the results [44]. After an adaptation period of 3 weeks, faeces collection started. Every day, half an hour after feeding, tanks were thoroughly cleaned to remove any uneaten pellets and a recipient, covered with ice packs, was inserted at the water outlet channel at the bottom of the tank to collect faeces by a settling decantation system. On the following day, faeces were collected from each tank and frozen at −20°C until analysis. Further, the faecal samples were analysed to indirectly determine the apparent digestibility coefficients (ADCs) of the dietary nutrients using yttrium oxide (Y_2_O_3_), an inert marker included in all feeds at 0.02%, according to the following formula:

ADCs (%) of dietary nutrients and energy [45]:

$\text{ADC }\left(\%\right)=100\times \left[1-\frac{\text{dietary marker }\left(\%\right)\;}{\text{faecal marker }\left(\%\right)}\times \frac{\text{faecal nutrient or energy level }}{\text{dietary nutrient or energy level }}\right].\;$ ]

ADC (%) of dry matter (DM):

$\text{ADC }\left(\%\right)=100\times \left[1\;-\frac{\text{dietary marker }\left(\%\right)\;}{\text{faecal marker }\left(\%\right)}\right].$ ]

### 2.5. Key Performance Indicators

Key performance indicators were calculated as follows:

  $$\begin{array}{c} \text{Weight gain (\% IBW; WG)}=100\times \text{wet weight gain }\left(g\right)\;\times \text{ initial biomass }{\left(g\right)}^{-1}, \end{array}$$

where wet weight gain (g) = final biomass (g) − initial biomass (g).

  $$\begin{array}{c} \text{Specific growth rate}\left(\;{\text{day}}^{-1};\text{ SGR}\right)=\;\left[\ln\left(\text{Final body weight }\left(g\right)\right)\;-\ln\left(\text{initial body weight }\left(g\right)\right)\;\times \text{ number of feeding }{\text{days}}^{-1}\right]. \end{array}$$

  $$\begin{array}{c} \text{Feed conversion ratio }\left(\text{FCR}\right)\;=\text{Apparent feed intake }\left(g\right)\;\times \text{ wet weight gain }{\left(g\right)}^{-1}. \end{array}$$

  $$\begin{array}{c} \text{Feed intake }\left(\text{g}/\text{fish};\text{ FI}\right)\;=\text{Feed distributed }\left(g\right)\;\times \text{ total number of }{\text{fish}}^{-1}. \end{array}$$

  $$\begin{array}{c} \text{Protein efficiency ratio }\left(\text{PER}\right)\;=\text{Wet weight gain }\left(g\right)\;\times \text{crude protein intake }{\left(\text{g DM}\right)}^{-1}. \end{array}$$

  $$\begin{array}{c} \text{Viscerosomatic index }\left(\%;\text{ VSI}\right)\;=\;100\;\times \text{viscera weight }\left(g\right)\;\times \text{body weight }{\left(g\right)}^{-1}. \end{array}$$

  $$\begin{array}{c} \text{Hepatosomatic index }\left(\%;\text{ HSI}\right)\;=\;100\;\times \text{liver weight }\left(g\right)\;\times \text{body weight }{\left(g\right)}^{-1}. \end{array}$$

  $$\begin{array}{c} \text{Condition factor }\left(K\right)=100\;\times \text{body weight }\left(g\right)\;\times \text{ total }{\text{length}}^{3}{\left(\text{cm}\right)}^{-1}. \end{array}$$

  $$\begin{array}{c} \text{Crude phosphorus intake }\left(\text{mg}.{\text{kg}}^{-1}.{\text{day}}^{-1};\text{ CPI}\right)\;=\;1000\times \left(\text{phosphorus intake }\left(\text{g DM}\right)\;\times \text{ biomass weight }{\text{(kg)}}^{-1}\;\times \text{number of feeding }{\text{days}}^{-1}\right). \end{array}$$

  $$\begin{array}{c} \text{Phosphorus }\left(\text{P}\right)\text{ gain }\left(\text{mg}.{\text{kg}}^{-1}.{\text{day}}^{-1};\text{ PG}\right)\;=\;1000\times \left(\text{final whole}-\text{body }P\text{ content }\left(\text{\% DM}\right)\;-\text{ initial whole}-\text{body }P\text{ content }\left(\text{\% DM}\right)\;\times \text{ biomass weight }{\left(\text{kg}\right)}^{-1}\;\times \text{number of feeding }{\text{days}}^{-1}\right). \end{array}$$

  $$\begin{array}{c} \text{Faecal phosphorus }\left(\text{P}\right)\text{ losses }\left(\text{mg}.{\text{kg}}^{-1}.{\text{day}}^{-1};\text{ FPL}\right)\;=\text{ Crude P intake }\left(\text{mg}.{\text{kg}}^{-1}.d^{-1}\right)\;\times \text{ apparent digestibayility coefficient of }P\left(\text{\%}\right). \end{array}$$

  $$\begin{array}{c} \text{Metabolic phosphorus }\left(\text{P}\right)\text{ losses }\left(\text{mg}.{\text{kg}}^{-1}.{\text{day}}^{-1};\text{ MPL}\right)\;=\text{ Crude }P\text{ intake }\left(\text{mg}.{\text{kg}}^{-1}.{day}^{-1}\right)\;-\;P\text{ gain }\left(\text{mg}.{\text{kg}}^{-1}.{day}^{-1}\right)\;+\text{ faecal }P\text{ losses }\left(\text{mg}.{\text{kg}}^{-1}.{day}^{-1}\right). \end{array}$$

  $$\begin{array}{c} \begin{array}{c} \;\times \;\left(\text{final whole}-\text{ body protein},\text{ lipid},\text{phosphorus or energy content }-\text{ initial whole}\right.\\ \;\left.-\text{ body protein},\text{ lipid},\text{phosphorus or energy content}\right)\\ \;\times \;\left(\text{crude protein},\text{crude lipid},\text{phosphorus or gross energy }{\text{intake}}^{-1}\right.\\ \left.\;\times \text{ ADC\% of protein},\text{ lipid},\text{phosphorus or energy}\right). \end{array} \end{array}$$

### 2.6. Analytical Procedures

Diet samples, faeces and whole-fish were freeze dried and ground until a homogeneous powder was obtained. Chemical analyses were made in duplicates and following the methodology described by AOAC [46]: dry matter after drying at 105°C for 24 h (method 934.01); total ash by combustion (550°C during 12 h) in a muffle furnace (L9/11/B170, Nabertherm, Lilienthal, Germany; method 942.05); crude protein (*N* × 6.25) by a flash combustion technique followed by a gas chromatographic separation and thermal conductivity detection with a Leco N Analyzer (Model FP-528, Leco Corporation, St. Joseph, MI, USA; method 990.03); crude lipid by petroleum ether extraction (40–60°C) using a Soxtec 2055 Fat Extraction System (Foss, Hillerod, Denmark), with prior acid hydrolysis with 8.3 M HCl (method 954.02); gross energy in an adiabatic bomb calorimeter (Werke C2000, IKA, Staufen, Germany).

Phosphorus concentrations in diets, faeces and whole-fish as well as yttrium concentrations in diets and faeces were initially determined by weighting (50–125 mg) dry samples in quartz vessels. Samples were then digested in 6 mL of nitric acid (HNO_3_ tracer grade, 70%) in a Discovery SP-D microwave digestion unit (CEM, Matthews, NC, USA) according to the following programme: 200°C; 4 min ramp; 3 min hold. The samples were then cooled to room temperature and a final volume of 10 mL was achieved by adding ultrapure water. Subsequently, samples were diluted in ultrapure water and the standard curves prepared. Mineral quantification was performed by MP-AES (model 4200, Agilent, Santa Clara, CA, USA) at 371 nm for phosphorus and 214 nm for yttrium. Blank samples containing only the decomposition acid were included to measure the matrix effects of decomposition, which were subtracted from every element in each sample.

### 2.7. Reverse Transcription–Quantitative Real-Time PCR (qRT-PCR)

Samples from the anterior intestine of three fish per replicate tank (*n* = 3 tanks per dietary treatment per experimental period) were collected and analysed at the end of both growth and challenge periods. Total RNA was extracted using the Maxwell RSC simplyRNA Tissue Kit (Promega Corporation, Madison, WI, USA) according to the manufacturer's instructions. Total RNA quality and integrity was determined by denaturing agarose gel electrophoresis, while concentration and purity were based on absorbance at 260 nm and ratios at 260:280 and 260:230 nm, using a Nanodrop OneC (Thermo Fisher Scientific, Waltham, MA, USA). Complementary DNA (cDNA) synthesis was performed by reverse transcription of 1000 ng of total RNA using the RevertAid H Minus First Strand Kit (Thermo Fisher Scientific), according to the manufacturer's protocol. Real-time PCR (RT-PCR) was performed in a CFX384 Real Time PCR detection system (Bio-Rad, Hercules, CA, USA) with PowerTrack SYBR Green chemistry (Thermo Fisher Scientific), using specific primers (Table 3). Primers for each gene were designed using the Geneious Prime version 2023.1 (https://www.geneious.com Geneious, Boston, MA, USA) based on sequences from the GenBank database (NCBI, Bethesda, MD, USA) [47]. Biomarkers for the oxidative status include superoxide dismutase (*sod*), catalase (*cat*), glutathione peroxidase (*gpx*) and nuclear factor erythroid 2-related factor 2 (*nrf2*). Regarding immune condition, interleukin-1*β* (*il-1β*), immunoglobin (*igm*) and cyclooxygenase-2 (*cox2*) were analysed. The intestinal epithelial integrity was assessed for mucin 13 (*muc13*), claudin 12 (*cldn12*), tight junction protein 2 (*tjp2*), occludin (*ocl*) and proliferating cell nuclear antigen (*pcna*). The RT-PCR assays were run in duplicates in a 10 μL volume containing 2 μL of cDNA, 0.625 μL of each specific forward and reverse primers at 10 μM, 5 μL of PowerTrack SYBR Green Master Mix (Thermo Fisher Scientific) and 1.75 μL of nuclease-free water. The amplification protocol was set as follows: an initial denaturation step of 2 min at 95°C, followed by 40 cycles of denaturation for 5 sec at 95°C and 30 sec at 55, 57 or 59°C for annealing/extension, depending on the primer specificity. Negative controls without sample templates were consistently executed for each primer set. The specificity of reactions was confirmed through the examination of melting curves, using ramping rates of 0.5°C/5 s, across a temperature span of 60–95°C. Gene expression levels were normalised using the geomean from two reference housekeeping genes, elongation factor 1 *α* (*ef1-α*) and 18S ribosomal RNA (*18S*). The relative mRNA expression of the target genes was calculated according to the Pfaffl method [48]. The relative gene expressions of fish fed each diet were analysed at the end of the growth and challenge periods. At the end of the growth period, gene expressions were calculated using the CTRL dietary treatment as reference. To compare the gene expressions pre- and post-stress, the relative expressions at the end of the growth period were used as reference.

### 2.8. Data Analysis and Statistics

All results are expressed as mean ± standard deviation (mean ± SD). Data were checked for normality and homogeneity of variances with Shapiro–Wilk and Levene's test, respectively. Results expressed as percentages (viscerosomatic index [VSI], hepatosomatic index (HSI), survival, ADCs, whole-body composition and retentions) were, prior to statistical analysis, transformed using arcsine square root. Relative gene expression data were transformed by a Box–Cox transformation. When conditions were met, a one-way ANOVA followed by Tukey's multiple-comparison test were used to identify differences among groups. If conditions were not verified, a non-parametric Kruskal–Wallis followed by a Dunn's post hoc pairwise comparison test were performed instead. To identify specific differences in the relative gene expression before and after the stress event, a one-way ANOVA planned contrasts analysis [49] was planned a priori. Here we planned the following contrasts: (1) Across all dietary treatments at the end of the growth and challenge periods, to identify which relative genes expressions were affected by the overcrowding event, regardless of the dietary treatment; (2, 3 and 4) Each dietary treatment independently (CTRL, ORG and ECO) at the end of the growth and challenge periods, to identify which genes' relative expression were significantly affected by the stress within each dietary treatment. To complement such analysis and understand the overall response of fish, we followed an integrative approach through an exploratory multivariate analysis. Here, we integrated the relative expression of biomarkers associated with oxidative status, immune condition, intestinal epithelium integrity and homeostasis in a principal component analysis (PCA) using RStudio (Boston, MA, USA). Since the PCA is an unsupervised exploratory technique, it was used to identify dataset underlying structures that could reveal if there was an association between overall response to stress patterns and diets, as well as to highlight the main variables that had the most influence on the data. Accordingly, for the PCA analysis, the standard *prcomp* function in R was applied to the auto-scaled matrices, while score plots for the first two principal components (PC1 and PC2) were generated using the *ggbiplot* and *factoextra* packages. PC1 and PC2 were chosen as the main principal components given that their eigenvalues accounted for most of the dataset variability. The score plots included confidence ellipses representing 95% confidence intervals around the centroid of each data cluster. The *fviz_cos2* function was used to view the quality of representation (*cos2*) of the variables in the principal components. The scores from the retrieved principal components were further analysed as new variables, which underwent a Box–Cox transformation and were subsequently analysed using a Student's *t* test to identify differences between the growth and challenge periods. The level of significance considered was *p* < 0.05 for all statistical tests. Statistical analyses were performed using the computer package IBM SPSS Statistics version 26.0 (Armonk, NY, USA) and RStudio version 4.2.

## 3. Results

### 3.1. Apparent Digestibility Coefficients of Diets

Nutrient and energy ADCs are presented in Table 4. Diet ORG showed significantly higher ADCs of protein (*p* = 0.001) and phosphorus (*p* = 0.023) compared to the other feeds. Dry matter (*p* = 0.516), lipids (*p* = 0.330) and energy (*p* = 0.999) were similar between ORG and CTRL. Diet ECO showed a similar digestibility to diet CTRL regarding protein (*p* = 0.066) and phosphorus (*p* = 0.985), while dry matter (*p* = 0.001), lipids (*p* = 0.001) and energy (*p* = 0.003) were significantly higher in CTRL.

### 3.2. Growth Performance, Feed Intake and Somatic Indices

At the end of the growth period, all fish increased their initial body weight at least three-fold (Table 5). There were no statistical differences regarding the specific growth rate (SGR; *p* = 0.061) among dietary treatments. Nevertheless, the final body weight (FBW) of fish fed ECO and CTRL diets were higher than ORG fish (*p* = 0.002). FI (*p* = 0.027) was lower in fish fed ORG compared to ECO fish, while there was a higher feed conversion ratio (FCR; *p* = 0.046) in fish fed ORG compared to CTRL and ECO fish (Table 5). The dietary treatments had no significant impact (*p* > 0.05) regarding the protein efficiency ratio (PER; *p* = 0.051), VSI (*p* = 0.245), HSI (*p* = 0.757) and condition factor (*K*; *p* = 0.174). Similarly, survival was high (>92%) and not affected by the dietary treatments (*p* = 0.109).

### 3.3. Fish Whole-Body Composition, Retentions and Phosphorus Balance

Regarding data on fish whole-body composition and retentions, most of the nutrients or energy showed no statistically significant differences among dietary treatments in the analysed parameters (Table 6). The only exception was phosphorus retention, which was significantly higher in fish fed ORG than those fed CTRL (*p* = 0.033).

**Figure 1:**
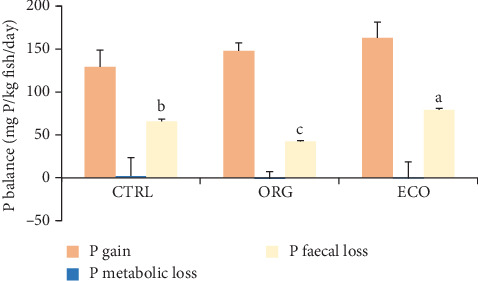
Daily phosphorus (P) balance (mg P/kg fish/day) in gilthead seabream (*Sparus aurata*), after 65 days of feeding (growth period) with three different experimental diets (CTRL, ORG and ECO). Data are presented as means ± standard deviation (*n* = 3). Different superscript letters indicate significant differences (one-way ANOVA; *p* < 0.001) between dietary treatments among the same fraction.

The daily phosphorus balance is presented in Figure 1. The dietary treatments only had a significant effect on the daily phosphorus faecal loss (*p* < 0.001). Fish fed the ECO diet had the highest faecal phosphorus losses (79.0 ± 1.99 mg P kg^−1^ day^−1^), followed by those fed with CTRL (65.8 ± 2.45 mg P kg^−1^ day^−1^), while fish fed ORG had the lowest values (42.5 ± 0.83 mg P kg^−1^ day^−1^). Phosphorus gain (*p* = 0.111) ranged from 163.0 ± 18.26 mg P kg^−1^ day^−1^ in the ECO treatment to 129.2 ± 19.62 mg P kg^−1^ day^−1^ in the CTRL treatment. Metabolic phosphorus losses were very small and comparable among diets (*p* = 0.972).

### 3.4. Molecular Biomarkers Analysis

Most of the molecular biomarkers at the end of the growth period were not significantly affected by the dietary treatments (Table 7). The only exception was immunoglobulin M (*igm*), where fish fed ORG and ECO showed an up-regulation (*p* < 0.001) when compared to those fed CTRL.

**Figure 2:**
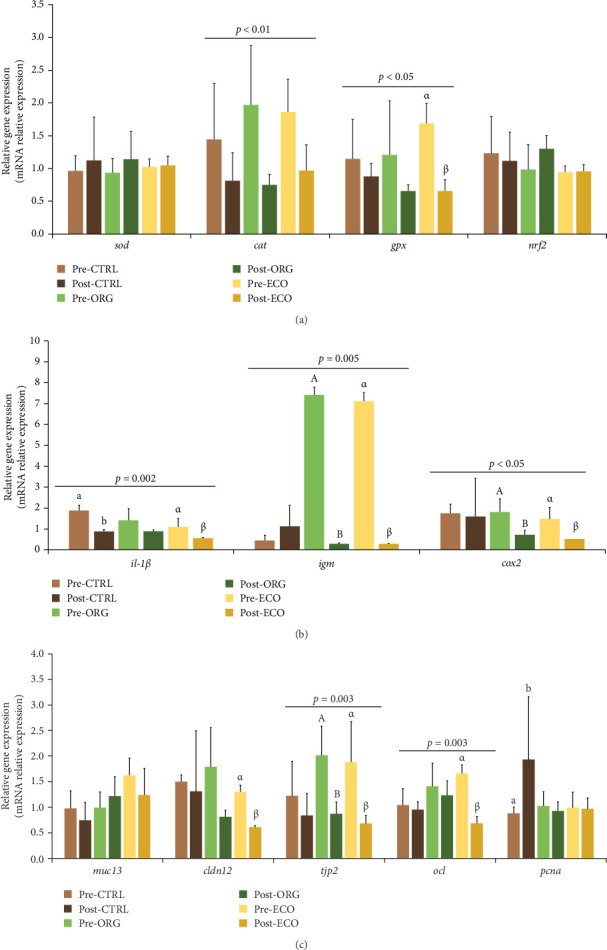
Relative expression (mRNA relative expression) of genes encoding for (a) oxidative status (*sod*, *cat*, *gpx* and *nrf2*), (b) immune condition (*il-1β*, *igm* and *cox2*) and (c) intestinal epithelium integrity (*muc13*, *cldn12*, *tjp2*, *ocl* and *pcna*) in the anterior intestine of gilthead seabream (*Sparus aurata*) juveniles, after feeding with three different experimental diets (CTRL, ORG and ECO), analysed pre- and post-stress. Data are presented as mean ± standard deviation (*n* = 3 tanks per dietary treatment). Different superscript letters (CTRL: a and b; ORG: A and B; ECO: α and β) indicate significant differences (planned contrasts one-way ANOVA; *p* < 0.05) between sampling periods (growth vs. challenge) among the same dietary treatment. Genes within lines on top with *p* value indicate significant differences (planned contrasts one-way ANOVA; *p* < 0.05) between sampling periods (growth vs. challenge) while considering all dietary treatments. Molecular biomarkers abbreviations same as in Table 7.

To analyse the effects of the challenge period, the planned contrasts for one-way ANOVA revealed that the overcrowding stressful event significantly affected the relative expression of several genes (Figure 2). The first contrast compared the expression of all genes in all dietary treatments pre and post stress, revealing that *cat* (*p* = 0.007), *gpx* (*p* = 0.033), *il-1β* (*p* = 0.002), *igm* (*p* = 0.005), *cox2* (*p* = 0.046), *tjp2* (*p* = 0.003) and *ocl* (*p* = 0.003) were significantly down-regulated following the challenge period. Fish fed the CTRL diet showed a down-regulation of *il-1β* (*p* = 0.002), while *pcna* was up-regulated (*p* = 0.033) following the challenge period. Fish fed ORG had *igm* (*p* = 0.001), *cox2* (*p* = 0.044) and *tjp2* (*p* = 0.031) significantly down-regulated when comparing the expression values between fish sampled at the end of the growth and challenge periods. Furthermore, *gpx* (*p* = 0.022), *il-1β* (*p* = 0.046), *igm* (*p* < 0.001), *cox2* (*p* = 0.017), *cldn12* (*p* = 0.001), *tjp2* (*p* = 0.0015) and *ocl* (*p* < 0.001) in fish fed ECO had their relative expression decreased after the stress event.

**Figure 3:**
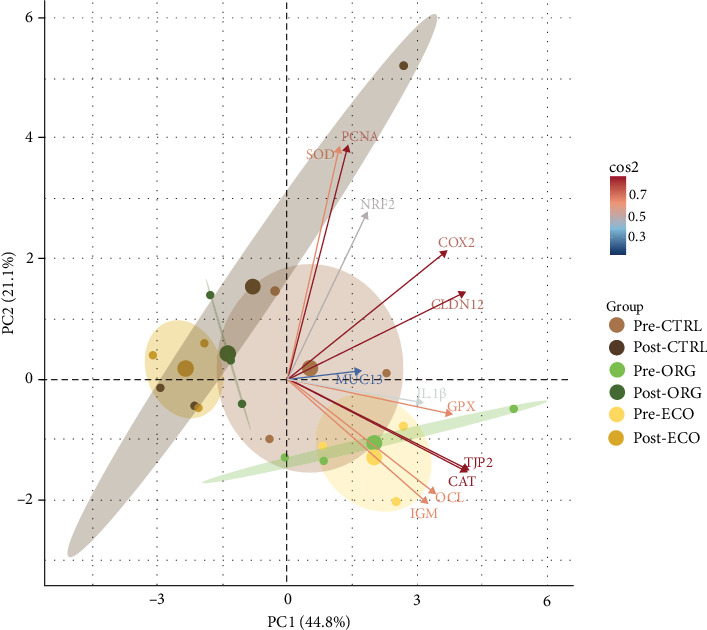
Score plot from the principal component analysis (PCA) based on the relative gene expression in the anterior intestine of gilthead seabream (*Sparus aurata*) juveniles, after feeding with three different experimental diets (CTRL, ORG and ECO), analysed pre- and post-stress. The first two principal components (PC1 and PC2) are shown on the axis. Scores were grouped by experimental diet (CTRL, ORG and ECO), pre- and post-stress. Each point represents the projection of an individual sample in the PC1 and PC2 axes. The ellipses represent 95% confidence intervals around the centroid (larger point) of each data cluster. Cos2 scale indicates variable loadings. Pre-CTRL, Post-CTRL, Pre-ORG, Post-ORG, Pre-ECO, Post-ECO: fish fed diet CTRL, ORG or ECO, respectively, at the end of the growth (pre-) or challenge (post-) period. Molecular biomarkers abbreviations same as in Table 7.

The PCA revealed that fish response was affected by the overcrowding environment (Figure 3). When assessing data structure through PCA, the PC1 and PC2 accounted for 65.9% of the dataset variability. The PC1 alone accounted for 44.8% and was strongly related with stress (e.g., before and after stress groups' pattern distinguished along PC1). This was loaded mainly by the lower relative expression of *cat*, *gpx*, *cox2*, *cldn12*, *tjp2* and *ocl* mainly from fish fed ORG and ECO. This differential response of fish before and after stress was confirmed with significant differences between the groups' scores (*p* = 0.02). When assessing the impact of each diet on the overall response pattern before and after the stress based on the scores retrieved for each group, fish fed CTRL or ORG diet had no differences, whereas fish fed ECO only had different scores before and after stress on PC1 (*p* = 0.005), but not on PC2 (*p* = 0.077).

## 4. Discussion

To attain a sustainable development, while ensuring the performance and physiological resilience of farmed organisms, aquaculture should formulate feeds considering alternative ingredients that improve the consumer perception of the industry. Additionally, these new formulations ideally should encompass ingredients that could potentially aid to decrease the sector environmental footprint. In this study, diet formulations based on organic or circular economy-derived frameworks were tested. At the end of the growth period and regardless of the diet, fish health was maintained, based on the relative gene expression and somatic indices, while achieving growth increases of at least three-fold. Although fish fed ORG showed lower growth, the VSI, HSI and *K* were similar to those from CTRL and ECO fish, suggesting a proper nutrition. Some of these values are slightly different from previous data on seabream fed alternative and organic-based diets [29, 50, 51]. However, in the present study, values were within the expected range and differences could be attributed to fish size, experimental conditions and feeding regimes [52, 53]. Furthermore, there was no clear evidence that the chronic overcrowding event negatively affected the overall fish stress response. The experimental feeds were highly digestible and no differences were observed in feed utilisation between experimental treatments. FI is usually regulated by feed proximal composition and palatability [54]. Feeds were formulated to fulfil the known species nutritional requirements, being isonitrogenous and isoenergetic. Moreover, although the fatty acid profile was not evaluated, the values of EPA + DHA, monounsaturated (MUFA) and total polyunsaturated fatty acids (PUFA) were all estimated and considered during formulation to fulfil the nutritional requirements in all diets. Accordingly, the potential effects of biochemical composition on the lower fish performance of fish fed ORG are unlikely. Therefore, feed palatability appears to be the most possible cause for the reduced intake.

Particularly the inclusion of plant ingredients, might have been the main responsible for the reduced ORG palatability, decreased intake and consequently lower growth. Compared with CTRL and ECO feeds, ORG had a higher amount of plant proteins. Although a reduction in FI and growth has been observed in gilthead seabream, European seabass (*Dicentrarchus labrax*), channel catfish (*Ictalurus punctatus*) and Atlantic salmon (*Salmo salar*) when fed with plant-rich feeds, only some of these results were linked to reduced palatability [55–60]. Conversely, a plant mixture did not alter or even increased intake and growth of juvenile seabream in other studies [29, 61, 62]. Different results can be attributed to differences in fish species, size, formulations, ingredient processing and inclusion levels of plants [50, 63]. Diet ORG could have had a bitter taste and astringent properties. Since fish can evaluate the organoleptic properties of feed (texture, flavour and odour), they might have found the overall sensory characteristics unpalatable, leading to the reduced FI [64–67]. In addition, the presence of ANFs (tannins, solanine, phytic acid, saponin and gossypol), as well as changes in pellet properties (e.g., hardness and colour) due to a higher inclusion of particular vegetables sources (e.g., spirulina, rapeseed and potato protein concentrate) could have also impacted feed palatability and intake [31–33, 56, 57, 64, 68, 69]. Regardless of the lower palatability of ORG, all experimental feeds were highly digestible.

All ADCs of the experimental feeds were considered to be high and normal for the species (particularly protein and lipid ADCs should be above 75% and 85%, respectively), meaning that the diets were highly digestible, with only ECO being less digestible than the other feeds, likely due to the inclusion of LAPs [70, 71]. Although LAPs often have a high nutritional value to fish, they can have some drawbacks mainly due to presence of connective tissue, skin and high keratin content, and could affect the activities of digestive enzymes, as they may contain protease and lipase inhibitors [72–76]. Some studies found that poultry meal are well tolerated by carnivorous fish species, despite they can exert some effects on the activities of digestive enzymes [74, 77–79]. Nengas et al. [75] reported that poultry meal, fed to gilthead seabream, had a high protein digestibility; however, when feather meal was included, protein digestibility decreased [75]. Campos et al. [80] showed that a higher inclusion of more than 7.5% of blood meal in the diets of European seabass decreased protein and energy ADCs [80]. Likewise, more than 4% blood meal inclusion decreased protein and lipid digestibility in juvenile red sea bream (*Pagrus major*) [81]. Therefore, it is more likely that the inclusion levels of poultry blood meal and feathermeal hydrolysate had a more pronounced role. However, results are always highly variable, as feed ADCs greatly depend on the inclusion levels of ingredients, dietary formulations, quality of raw materials and processing conditions during the rendering process (*e.g*., heat treatment, particle size and drum drying or spray drying) [77, 82]. Energy and dry matter ADCs were likely indirectly affected by protein and lipid ADCs and were in agreement with other studies using alternative formulations in fish species [57, 83–86]. Nevertheless, the observed ECO digestibility differences did not affect feed utilisation.

From the whole-body composition, and nutrient and energy retentions, only phosphorus retention differed between dietary treatments, which may suggest that fish physiological mechanisms efficiently utilised feed and metabolic functions remained unaffected by the dietary treatments. No changes in body composition were observed when alternative and eco-organic feeds were fed to gilthead seabream and red seabream [51, 87]. In the case of ECO, it is likely that fish compensated for the lower digestibility (although it was still high) through efficient metabolic processes and compensatory nutrient utilisation, providing sufficient nutrients and energy for optimum fish performance [77, 88]. Similarly, a previous study showed that a feed rich in LAPs also had lower protein digestibility, but showed increased retention, no effects on the whole-body composition of juvenile seabream and resulted in better fish growth than the control treatment [29]. Phosphorus retention of fish fed diet ORG was higher than CTRL fed fish, mainly due to the higher phosphorus ADC and consequent bioavailability of ORG [85, 89, 90]. Similarly to feed utilisation being unaffected by the dietary treatments, fish physiological resilience was also maintained.

At the end of the growth period, in agreement with overall performance, from all the assessed markers, only the expression of *igm* was affected by the dietary treatments, suggesting that the experimental feeds maintained fish health status. Immunoglobulin is a class of antibodies produced by B cells, that trigger and play a crucial role in early humoral immune regulation, tolerance and response against pathogens or environmental stress [91–93]. In this sense, an increase in expression of *igm* could suggest a stronger and enhanced adaptive immunity of fish fed ORG and ECO [94–96]. A study reported an upregulation of *igm* in the anterior intestine of juvenile seabream when fed a diet rich in LAPs [97]. In addition to the growth period, fish were exposed to a chronic challenge event.

After a chronic challenge, planned contrasts and PCA analysis revealed distinct responses in fish before and after the stress; however, an overall downregulation of the genes may be just a new allostatic equilibrium. In medium or long-term stressful situations, the relative expression of immune-related, antioxidant and epithelium integrity genes were affected and decreased in the intestine of common carp (*Cyprinus carpio*), darkbarbel catfish (*Pelteobagrus vachelli*), Atlantic salmon, rainbow trout (*Oncorhynchus mykiss*) and gibel carp (*Carassius gibel*) [98–102]. Stress is a immunosuppressor process in chronic stressful periods, hence fish have a higher energy demand that is primarily directed towards coping mechanisms [96, 103, 104, 105, 106, 107]. Therefore, it is common for the molecular machinery responsible for oxidative stress management, immune modulation and intestinal barrier integrity to reduce activity, as these processes are energy-demanding [94–96, 103, 108]. Planned contrasts indicated that fish fed ECO and ORG diets had more downregulated genes compared to fish fed CTRL, indicating a stronger downregulation following stress. At least in ECO feed, such differences could be related with the lower digestibility levels that could result in lower energy available in stressed fish. However, when assessing the overall response by integrating all markers using the PCA analysis, a significant difference was observed for PC1 (scores significantly different before and after stress), but not for PC2. In addition, fish fed CTRL and ORG also did not show any statistically significant differences in either PC1 or PC2. Nevertheless, the health measurement in the present study was only at the molecular level. Still, there was no indication that gene downregulation in fish fed ECO and ORG feeds reflected in a less resilient fish, particularly since fish continued to eat and grow, which suggests that seabream were coping effectively with the stress. In the future, incorporating disease response into similar studies would provide an additional perspective on fish health status [109, 110]. Besides fish resilience, some of the possible environmental impacts of aquafeeds were assessed.

Due to differences in feed composition, ORG and ECO feeds exhibited a higher GWP than CTRL, which could affect the environmental performance. ORG and ECO had higher inclusions of by-products and side streams (LAPs, salmon oil, brewer's yeast and microbial meal), algae (*Arthrospira platensis* and *Schyzochytrium*) and plant-based sources (e.g., potato protein concentrate, wheat gluten and corn gluten meal), along with the reduced inclusion of marine ingredients (fishmeal, fish protein hydrolysate and fish oil). Based on our analysis and according to the literature, LAPs, SCMs and plants can significantly contribute to carbon emissions due to their production (e.g., fertiliser usage and risk of deforestation), processing (e.g., rendering, grinding and drying) and transport [11, 12, 15, 111–113]. Atlantic Salmon, rainbow trout, European seabass and meagre (*Argyrosomus regius*) feeds richer in plants, had a higher GWP than others with a higher inclusion of marine resources [114, 115]. Other studies reported that marine ingredients have a remarkably low carbon footprint and are even considered to be among the most sustainable of all global fisheries [12, 116, 117]. This can be contradictory to some studies who reported a higher GWP from fishmeal rich feeds [118, 119]. However, comparing the environmental impacts of feeds and ingredients across studies can be subjective and challenging due to the lack of standardisation in LCAs and variability that arises from distinct methodologies, data collections, system boundaries, geographical locations, assumptions, allocations, formulations, species and production processes, all of which can result in distinct outcomes [11, 114, 118]. Although ORG and ECO showed a higher GWP, they may not necessarily bring a higher environmental impact, as a complete assessment of such impact should include many other variables (e.g., eutrophication, acidification, resource usage and land use) out of the scope of this study. Moreover, the GWP of LAPs, SCMs and other ingredients with high energy requirements, tends to decrease dramatically when clean and renewable energies, within the societal urge for decarbonisation, are used [11, 120, 121]. In addition, despite the higher GWP, it is relevant to note that the inclusion of LAPs and SCMs, could indirectly improve system-wide performance, valorise side streams and by-products from other industries, increase resource efficiency and reduce waste [11, 111, 122, 123]. In fact, the high phosphorus retention of fish fed ORG, could help mitigate phosphorus discharge into the environment, thereby reducing potential ecological issues [124].

## 5. Conclusions

This study showed that while the organic feed may slightly reduce fish performance, due to lower intake and palatability, likely caused by plant ingredients, it still allowed fish to increase their initial body weight three-fold, in line with the other dietary treatments. Despite the lower digestibility and high phosphorus faecal loss of the eco-efficient feed, all feeds were easily digestible and efficiently utilised. In addition, based on our measurements, metabolic functions and fish health remained unaffected throughout the growth period, while overall fish resilience to chronic stress was not affected. Although the novel feeds have a higher GWP associated, they may promote system-wide performance and their environmental footprint can be reduced depending on the use of renewable energy sources in ingredient production and sourcing of proximity ingredients. In fact, the organic feed tested can reduce phosphorus releases into the environment. While our results are promising it is important to note that there is room for improvement (e.g., palatability, digestibility and GWP) in such alternative formulation concepts and that our conclusions are based on the specific formulations and methodologies used in this study. Therefore, to provide a more holistic understanding of the effects of fish feeds, it would be interesting to evaluate these feed concepts in other species, with different experimental approaches (e.g., acute stress) and a more comprehensive environmental assessment that includes a wider range of environmental impacts (e.g., eutrophication and resource usage). Nevertheless, our study points that the organic and circular economy-derived feeds have potential to mitigate some of the environmental impacts of aquaculture, without compromising fish performance and physiological resilience.

## Figures and Tables

**Table 1:** Diet formulation (% inclusion levels), proximate composition (% as fed) and global warming potential (GWP; kg CO_2_ eq/tonnes feed) of the experimental diets (CTRL, ORG and ECO) for gilthead seabream (*Sparus aurata*).

Ingredients (% inclusion levels)	CTRL	ORG	ECO
Fishmeal super prime^a^	15.00	15.00	—
Fishmeal^b^	5.00	—	5.00
Fish protein hydrolysate^c^	3.00	—	3.00
Poultry meal^d^	15.00	—	20.00
Poultry blood meal^e^	3.00	—	5.00
Feathermeal hydrolysate^f^	5.00	—	10.00
Microbial meal^g^	4.00	—	4.00
Brewer's yeast^h^	—	5.00	—
*Arthrospira platensis* ^i^	—	5.00	—
Potato protein concentrate^j^	—	8.90	—
Pea protein concentrate^k^	—	11.00	—
Wheat gluten^l^	—	11.00	—
Corn gluten meal^m^	8.00	—	5.70
Guar korma^n^	5.00	9.50	5.00
Rapeseed meal^o^	—	4.50	—
Sunflower meal^p^	3.00	—	6.00
Wheat meal^q^	13.48	7.58	12.63
Whole peas^r^	5.50	5.50	5.50
Vitamin and mineral premix^s^	1.00	1.00	1.00
Choline chloride^t^	0.20	0.20	0.20
Antioxidant^u^	0.20	0.20	0.20
Mono-calcium phosphate^v^	—	1.10	1.65
L-Lysine^w^	—	—	0.50
DL-Methionine^x^	—	—	0.05
Yttrium oxide^y^	0.02	0.02	0.02
Algae meal (*Schyzochytrium* spp)^z^	—	—	1.30
Rapeseed lecithin^aa^	0.50	0.50	0.50
Fish oil^bb^	4.45	7.00	4.45
Salmon oil^cc^	8.65	—	8.30
Rapeseed oil^dd^	—	7.00	—
Total	100	100	100
Proximate composition (% as fed)	CTRL	ORG	ECO
Dry matter (DM)	94.31	96.91	95.56
Ash	7.76	7.03	5.76
Crude protein	50.61	51.05	51.40
Crude fat	17.66	17.93	17.80
Total phosphorus	1.10	1.02	1.31
Gross energy (kJ/g^−1^)	21.80	22.2	22.54
GWP (kg CO_2_ eq/tonnes feed)	1407	1961	1550

*Note:* Proximate composition values are reported as mean of duplicate analyses.

^a^Fishmeal Super Prime, Diamante: 66.3% CP, 11.5% CF; Pesquera Diamante, Lima, Peru.

^b^Fishmeal, CONRESA: 61.2% CP, 8.4% CF; Conserveros Reunidos S.A., Aguiño, Spain.

^c^Fish protein hydrolysate, CPSP90: 82.6% CP, 9.6% CF; Sopropêche, Wimille, France.

^d^Poultry meal: 62.4% CP, 12.5% CF; SAVINOR UTS, Trofa, Portugal.

^e^Poultry blood meal: 90.0% CP, 1.0% CF, ECB COMPANY SRL A S.U, Treviglio, Italy.

^f^Feathermeal hydrolysate EM'PAQ: 88.8% CP, 1.6% CF; Empro Europe, Dendermonde, The Netherlands.

^g^Microbial meal (*Corynebacterium glutamicum*), Aminopro NT70: 74.1% CP, 3.1% CF, MAZZOLENI SPA, Bergamo, Italy.

^h^Brewer's yeast: 38.9% CP, 4.5% CF; Premix Lda, Neiva, Portugal.

^i^ *Arthrospira platensis*: 72.1% CP, 1.0% CF, Sopropêche, Wimille, France.

^j^Potato protein concentrate, Protamyl: 77.0% CP, 1.4% CF, AVEBE, Veendam, The Netherlands.

^k^Pea protein concentrate, Lysamine GPS: 78.1% CP, 8.3% CF, Roquette, Lestrem, France.

^l^Wheat gluten, VITAL: 80.4% CP, 5.8% CF, Roquette, Lestrem, France.

^m^Corn gluten meal: 61.2% CP, 5.2% CF, COPAM, São João da Talha, Portugal.

^n^Guar korma, Seah International, Wimille, France.

^o^Solvent extracted rapeseed meal: 34.3% CP, 2.1% CF, Ribeiro & Sousa Lda., Leiria, Portugal.

^p^Solvent extracted dehulled sunflower meal, HiPro: 42.9% CP, 3.8% CF, AGP Slovakia, s.r.o, Komárno, Slovakia.

^q^Wheat meal: 11.7% CP, 1.6% CF, Molisur, Sevilla, Spain.

^r^Whole peas: 19.6% CP, 2.2% CF, Ribeiro & Sousa Lda., Leiria, Portugal.

^s^Vitamin and mineral premix, WISIUM MIX AQUA 1.5%: PREMIX Lda, Neiva, Portugal. Vitamins (IU or mg/Kg diet): DL-alphatocopherol acetate, 100 mg; sodium menadione bisulphate, 25 mg; retinyl acetate, 20,000 IU; DL-cholecalciferol, 2000 IU; thiamine, 30 mg; riboflavin, 30 mg; pyridoxine, 20 mg; cyanocobalamin, 0.1 mg; nicotidin acid, 200 mg; folic acid, 15 mg; ascorbic acid, 1000 mg; inositol, 500 mg; biotin, 3 mg; calcium panthotenate, 100 mg; choline chloride, 1000 mg, betaine, 500 mg. Minerals (g or mg/kg diet): cobalt carbonate, 0.65 mg; copper sulphate, 9 mg; ferric sulphate, 6 mg; potassium iodide, 0.5 mg; manganese oxide, 9.6 mg; sodium selenite, 0.01 mg; zinc sulphate. 7.5 mg; sodium chloride, 400 mg; calcium carbonate, 1.86 g; excipient wheat middlings.

^t^Choline chloride 50%: ORFFA, Breda, The Netherlands.

^u^Antioxidant, VERDILOX: Kemin Europe NV, Herentals, Belgium.

^v^Mono-calcium phosphate, ALIPHOS MONOCAL: 22.7% P, 17.5% Ca, ALIPHOS, Louvain-la-neuve, Belgium.

^w^L-Lysine 99%: Ajinomoto EUROLYSINE S.A.S, Paris, France.

^x^DL-Methionine 99%: Rhodimet NP99, ADISSEO, Paris, France.

^y^Yttrium oxide, Amperit: Höganäs Germany GmbH, Dusseldorf, Germany.

^z^Algae meal (*Schizochytrium* spp.): 11% CP, 49.4% CL, 16% DHA, Allmicroalgae, Pataias, Portugal.

^aa^Rapeseed lecithin, CANOLECITHIN F60:94% CL, Novastell, Vernon, France.

^bb^Fish oil: 98.1% CL, 16% EPA; 12% DHA, Sopropêche, Wimille, France.

^cc^Salmon oil: 98.3% CL, 4.6% EPA; 5.2% DHA, Sopropêche, Wimille, France.

^dd^Rapeseed oil: 98.2% CL, JC Coimbra, Setúbal, Portugal.

**Table 2:** Amino acid composition (mg AA g^−1^ dry weight) of the experimental diets (CTRL, ORG and ECO) for gilthead seabream (*Sparus aurata*).

Amino acids (mg AA g^−1^ dry weight)	CTRL	ORG	ECO
Essential amino acids			
Arginine	31.20	33.79	33.01
Histidine	11.26	11.60	10.97
Lysine	26.33	29.11	27.86
Threonine	23.82	21.23	24.76
Isoleucine	21.86	24.23	22.08
Leucine	39.16	38.54	39.47
Valine	25.84	26.66	27.77
Methionine	8.61	10.27	10.31
Phenylalanine	22.78	25.25	23.66
Total	210.86	220.68	219.89
Non-essential amino acids			
Cystine	8.42	8.93	10.94
Tyrosine	15.91	19.91	18.17
Aspartic acid + asparagine	38.34	44.84	37.48
Glutamic acid + glutamine	71.44	94.45	70.44
Alanine	29.29	24.96	28.91
Glycine	33.14	27.15	34.78
Proline	30.36	31.40	33.61
Serine	25.02	25.13	30.65
Taurine	1.95	0.97	1.64
Total	253.87	277.74	266.82

*Note:* All values are reported as mean of duplicate analyses.

**Table 3:** Sequences of forward and reverse primers, along with their accession number and qRT-PCR efficiency (%) for the reference genes (*ef1-α* and *18S*) and molecular biomarkers indicators of the oxidative status, immune condition and intestinal epithelium integrity, used in the present study.

Gene	Forward primer sequence (5′ → 3′)	Reverse primer sequence (5′ → 3′)	NCBI GenBank accession number	qRT-PCR efficiency (%)
*sod*	TCACAGGAGAAATCAAAGGGCT	GGACCGCCATGATTCTTACCAT	JQ308832	128.6
*cat*	CGACATGGTGTGGGACTTCT	CGCTCACCATTGGCATTGAC	JQ308823	121.2
*gpx*	TTTACGCCCTGACAGCCAAT	AGTAACGACTGTGGAGCTCG	KC201352	113.4
*nrf2*	TGAAGGAGGAGAAGGAGCGT	AGTACTCGGACGGCGAGTAT	XM_030427725	111.3
*il-1β*	TCCAAGCTTGCATCTGGAGG	GCTGAAGGGAACAGACACGA	AJ277166	96.5
*igm*	GACAACCTCAGCGTCCTTCA	CTTTTGAGTCTGCAGCGTCG	JQ811851	117.8
*cox2*	GACATCATCAACACTGCCTCC	GATATCACTGCCGCCTGAGT	AM296029	120.0
*muc13*	CTGTCTACTGAACGGGGCAA	ATTCTGTCACTGAACGCCGT	JQ277713	109.9
*cldn12*	AGCCGTATTTGCCTGTCCAG	CGTAACTTTGTGAGGGGGCA	XM_030393069.1	101.0
*tjp2*	CTGCTGGATGTGACACCCAA	GGCGATCCTCTGTCTCAAGG	XM_030417304.1	107.8
*ocl*	TACGGTGGAATCGGAGGGAA	CTGGTGAGACACGACGATGA	JQ692876	108.1
*pcna*	TCATGATCTCCTGCGCCAAG	CAAAGATCAGCTGGACGGGT	KF857335	119.0
*ef1-α*	GGAGATGCACCACGAGTCTC	GCGTTGAAGTTGTCAGCTCC	AF184170	110.9
*18S*	TGCAGAATCCTCGCCAGTAC	GGTGAGCCCGGATCTTCTTC	AM490061	114.5

*Note:* 18S, 18S ribosomal RNA.

Abbreviations: cat, catalase; cldn12, claudin 12; cox2, cyclooxygenase-2; ef1-α, elongation factor 1 α; gpx, glutathione peroxidase; igm, immunoglobin; il-1β, interleukin-1β; muc13, mucin 13; nrf2, nuclear factor erythroid 2-related factor 2; ocl, occludin; pcna, proliferating cell nuclear antigen; sod, superoxide dismutase; tjp2, tight junction protein 2.

**Table 4:** Apparent digestibility coefficients (ADCs; %) of nutrients and energy of experimental diets (CTRL, ORG and ECO) given to gilthead seabream (*Sparus aurata*) during the growth and challenge periods.

Parameters	Diets			*p* value
	CTRL	ORG	ECO	
Dry matter (DM; %)	80.6^a^ ± 3.53	79.2^a^ ± 3.10	61.8^b^ ± 9.85	0.003
Protein (%)	94.7^b^ ± 1.12	96.6^a^ ± 0.52	89.6^b^ ± 2.86	0.001
Lipids (%)	98.5^a^ ± 0.28	98.3^a^ ± 0.20	96.4^b^ ± 0.71	0.002
Phosphorus (%)	66.6^b^ ± 8.95	77.5^a^ ± 2.77	67.2^b^ ± 7.76	0.023
Energy (%)	94.0^a^ ± 1.31	94.3^a^ ± 0.69	86.4^b^ ± 3.73	0.003

*Note:* Data are presented as mean ± standard deviation (*n* = 3). Different superscript letters within the same row indicate significant differences (Kruskal–Wallis; *p* < 0.05) between dietary treatments.

**Table 5:** Growth performance, feed intake and somatic indices of gilthead seabream (*Sparus aurata*), after 65 days of feeding (growth period) with three different experimental diets (CTRL, ORG and ECO).

Parameters	Diets			*p* value
	CTRL	ORG	ECO	
FBW (g)	47.8^a^ ± 1.43	42.6^b^ ± 0.32	48.8^a^ ± 1.62	0.002
SGR (day^−1^)	0.019 ± 0.001	0.017 ± 0.000	0.019 ± 0.000	0.061
FI (g/fish)	36.3^a,b^ ± 0.91	34.7^b^ ± 0.83	38.3^a^ ± 0.47	0.027
FCR	1.1^b^ ± 0.06	1.2^a^ ± 0.03	1.1^b^ ± 0.04	0.046
PER	1.9 ± 0.10	1.6 ± 0.04	1.9 ± 0.07	0.051
VSI (%)	7.7 ± 0.71	8.2 ± 0.16	7.6 ± 0.27	0.245
HSI (%)	1.1 ± 0.05	1.2 ± 0.11	1.2 ± 0.02	0.757
*K*	1.4 ± 0.03	1.5 ± 0.04	1.5 ± 0.02	0.174
Survival (%)	96.7 ± 0.10	96.7 ± 1.92	92.9 ± 3.51	0.109

*Note:* Data are presented as mean ± standard deviation (*n* = 3 replicates per dietary treatment). Different superscript letters within the same row indicate significant differences (one-way ANOVA; *p* < 0.05) between dietary treatments. *K*, condition factor.

Abbreviations: FBW, final body weight; FCR, feed conversion ratio; FI, feed intake; HSI, hepatosomatic index, PER, protein efficiency ratio; SGR, specific growth rate; VSI, viscerosomatic index.

**Table 6:** Whole-body composition (% wet weight) and retention of nutrients and energy (% digestible intake) in gilthead seabream (*Sparus aurata*), after 65 days of feeding (growth period) with three different experimental diets (CTRL, ORG and ECO).

Parameters	Initial	Diets			*p* value
		CTRL	ORG	ECO	
Whole body composition (% wet weight)					
Dry matter (DM; %)	26.8 ± 1.09	29.5 ± 0.23	30.2 ± 0.79	30.1 ± 0.39	0.308
Protein (%)	14.9 ± 0.57	15.3 ± 0.39	15.8 ± 0.20	15.8 ± 0.17	0.066
Lipid (%)	7.0 ± 0.34	8.7 ± 0.65	9.3 ± 0.49	8.9 ± 0.38	0.428
Ash (%)	3.9 ± 0.01	4.2 ± 0.42	4.4 ± 0.32	4.2 ± 0.15	0.697
Phosphorus (%)	0.9 ± 0.01	0.8 ± 0.09	1.0 ± 0.05	1.0 ± 0.07	0.073
Energy (kJ/g)	5.8 ± 0.08	6.5 ± 0.18	6.9 ± 0.32	6.7 ± 0.13	0.208
Retention (% digestible intake)					
Protein (%)	—	28.1 ± 2.07	25.8 ± 0.88	27.8 ± 1.56	0.061
Lipid (%)	—	48.9 ± 6.43	47.1 ± 2.42	48.2 ± 1.61	0.434
Phosphorus (%)	—	65.4^b^ ± 10.31	78.6^a^ ± 7.59	67.4^a,b^ ± 6.82	0.033
Energy (%)	—	28.7 ± 2.20	27.2 ± 1.57	27.7 ± 1.42	0.362

*Note:* Data are presented as mean ± standard deviation (*n* = 3 pools per dietary treatment). Different superscript letters within the same row indicate significant differences (one-way ANOVA or Kruskal–Wallis; *p* < 0.05) between dietary treatments.

**Table 7:** mRNA relative expression of genes of the anterior intestine of gilthead seabream (*Sparus aurata*) juveniles, after 65 days of feeding (growth period) with three different experimental diets (CTRL, ORG and ECO).

Target genes	Diets			*p* value
	CTRL	ORG	ECO	
Oxidative status (mRNA relative expression)				
* sod*	1.0 ± 0.25	1.0 ± 0.24	1.1 ± 0.13	0.771
* cat*	1.5 ± 0.87	2.0 ± 0.92	1.9 ± 0.51	0.682
* gpx*	0.9 ± 0.47	0.9 ± 0.64	1.3 ± 0.24	0.538
* nrf2*	1.1 ± 0.52	0.9 ± 0.35	0.9 ± 0.09	0.835
Immune condition (mRNA relative expression)				
* il-1β*	1.0 ± 0.14	0.8 ± 0.32	0.6 ± 0.22	0.166
* igm*	0.8^b^ ± 0.47	13.8^a^ ± 0.70	13.2^a^ ± 0.80	<0.001
* cox2*	1.1 ± 0.27	1.1 ± 0.40	0.9 ± 0.34	0.742
Epithelium integrity (mRNA relative expression)				
* muc13*	1.1 ± 0.38	1.1 ± 0.33	1.8 ± 0.37	0.107
* cldn12*	1.2 ± 0.11	1.4 ± 0.62	1.0 ± 0.11	0.699
* tjp2*	1.1 ± 0.63	1.8 ± 0.53	1.7 ± 0.73	0.369
* ocl*	1.2 ± 0.37	1.6 ± 0.51	1.9 ± 0.20	0.157
* pcna*	1.1 ± 0.15	1.2 ± 0.34	1.2 ± 0.37	0.784

*Note:* Data are presented as mean ± standard deviation (*n* = 3 tanks per dietary treatment). Different superscript letters indicate significant differences (one-way ANOVA; *p* < 0.05) between dietary treatments.

Abbreviations: *cat*, catalase; *cldn12*, claudin 12; *cox2*, cyclooxygenase-2; *gpx*, glutathione peroxidase; *igm*, immunoglobin; *il-1β*, interleukin-1*β*; *muc13*, mucin 13; *nrf2*, nuclear factor erythroid 2-related factor 2; *ocl*, occludin; *pcna*, proliferating cell nuclear antigen, *sod*, superoxide dismutase; *tjp2*, tight junction protein 2.

## Data Availability

The data that support the findings of this study are openly available in Zenodo at https://zenodo.org/.
